# Effect of caloric restriction on gut permeability, inflammation markers, and fecal microbiota in obese women

**DOI:** 10.1038/s41598-017-12109-9

**Published:** 2017-09-20

**Authors:** Beate Ott, Thomas Skurk, Ljiljana Hastreiter, Ilias Lagkouvardos, Sandra Fischer, Janine Büttner, Teresa Kellerer, Thomas Clavel, Michael Rychlik, Dirk Haller, Hans Hauner

**Affiliations:** 1 Else Kröner-Fresenius-Center of Nutritional Medicine, Technical University of Munich, Freising, Germany; 2 ZIEL Institute for Food and Health, Technical University of Munich, Freising-Weihenstephan, Germany; 3 Charité-Universitätsmedizin, Medizinische Klinik mit Schwerpunkt Hepatologie und Gastroenterologie, Berlin, Germany; 4 Institute of Medical Microbiology, RWTH University Hospital, Aachen, Germany; 5 Chair of Analytical Food Chemistry, Technical University of Munich, Freising, Germany; 6 Chair of Nutrition and Immunology, Technical University of Munich, Freising-Weihenstephan, Germany; 7 Institute of Nutritional Medicine, Klinikum rechts der Isar, Technical University of Munich, Munich, Germany

## Abstract

Recent findings suggest an association between obesity, loss of gut barrier function and changes in microbiota profiles. Our primary objective was to examine the effect of caloric restriction and subsequent weight reduction on gut permeability in obese women. The impact on inflammatory markers and fecal microbiota was also investigated. The 4-week very-low calorie diet (VLCD, 800 kcal/day) induced a mean weight loss of 6.9 ± 1.9 kg accompanied by a reduction in HOMA-IR (Homeostasis model assessment-insulin resistance), fasting plasma glucose and insulin, plasma leptin, and leptin gene expression in subcutaneous adipose tissue. Plasma high-molecular weight adiponectin (HMW adiponectin) was significantly increased after VLCD. Plasma levels of high-sensitivity C-reactive protein (hsCRP) and lipopolysaccharide-binding protein (LBP) were significantly decreased after 28 days of VLCD. Using three different methods, gut paracellular permeability was decreased after VLCD. These changes in clinical parameters were not associated with major consistent changes in dominant bacterial communities in feces. In summary, a 4-week caloric restriction resulted in significant weight loss, improved gut barrier integrity and reduced systemic inflammation in obese women.

## Introduction

A chronic positive energy balance leads to obesity and low-grade inflammation. This subclinical inflammatory state is considered to pave the way for insulin resistance and subsequent type 2 diabetes mellitus^1,2^ and to promote cardiovascular diseases^3^. However, the reasons for this low-grade inflammation are poorly understood. It may, in part, be explained by macrophage infiltration into adipose tissue^4,5^. Furthermore, adipocyte hypertrophy and the associated dysregulated adipokine secretion profile may contribute to inflammation^6^. Recent studies suggested that increased gut permeability^7^ and lipopolysaccharides (LPS) translocation may also play an important role^8,9^. Some evidence exists that BMI is a major covariate of microbiome variations and that obesity is associated with changes in intestinal microbiota composition^10–13^. A disrupted gut barrier function may lead to an increased influx of bacterial components such as LPS^9,14,15^ and other complex antigens into the circulation and, thereby, activate immune responses^8,16^. It is currently speculated that increased gut permeability can be caused by microbial imbalance, food composition (e.g. gluten)^17,18^ or high-calorie diets^19,20^. In contrast, weight reduction is known to improve metabolic disturbances and to decrease the systemic inflammatory tone^21,22^.

Based on the observation that obesity is associated with a chronic low-grade inflammatory state and, potentially, impaired gut permeability^7^, the present study aimed to investigate whether caloric restriction is able to modulate gut permeability and, thereby, decrease markers of inflammation in human subjects. Moreover, gut microbiota diversity and composition, and their possible association with gut permeability and inflammation was investigated.

## Results

### The calorie-restricted formula diet induced weight loss

Twenty women (mean age 46.8 ± 11.5) were included in the study and no participant dropped out. Physical and biochemical characteristics of the participants before intervention, immediately after the hypocaloric diet, and 14d after intervention are shown in Table 1. Participants were asked to consume a formula diet (800 ﻿kcal/day) ﻿and, in addition, 200 g vegetables. Compared to the original caloric intake of 1,698.1 ± 592.7 kcal/day, this intervention caused a substantial energy deficit (P < 0.0001).

**Table 1:** Anthropometric and metabolic characteristics of study participants. Data are presented as mean ± standard deviation. P-value for differences between time points before and after VLCD was determined using paired Student’s test.

	before VLCD (n = 20)	after VLCD (n = 20)	14d after VLCD (n = 20)	p - value
	t1	t2	t3	t1−t2
Weight (kg)	95.1 ± 13.4	88.2 ± 12.3	88.5 ± 12.6	<0.001
BMI (kg/m²)	34.9 ± 3.8	32.5 ± 3.5	32.6 ± 3.8	<0.001
Waist circumference (cm)	106.9 ± 10.6	101.2 ± 9.4	100.5 ± 9.3	<0.001
Hip circumference (cm)	118.5 ± 12.6	113.9 ± 10.8	114.4 ± 11.5	<0.001
Lean mass (kg)	52.7 ± 5.7	50.0 ± 5.5	50.9 ± 5.3	<0.001
Fat mass (kg)	42.5 ± 8.8	38.2 ± 7.9	37.6 ± 8.4	<0.001
RMR (kJ/day)	7,016 ± 109	6,689 ± 707	7,061 ± 716	0.07
Total cholesterol (mmol/L)	4.9 ± 1.0	4.2 ± 0.8	4.8 ± 0.9	<0.001
HDL-c (mmol/L)	1.3 ± 0.2	1.1 ± 0.2	1.3 ± 0.3	<0.001
LDL-c (mmol/L)	3.1 ± 0.9	2.6 ± 0.7	2.9 ± 0.8	<0.001
Triglycerides (mmol/L)	1.4 ± 0.7	1.1 ± 0.5	1.3 ± 0.7	0.05
LDL/HDL	2.4 ± 0.8	2.3 ± 0.7	2.2 ± 0.7	0.68
NEFA (mmol/L)	0.6 ± 0.2	0.7 ± 0.2	0.5 ± 0.2	0.03
Fasting glucose (mmol/L)	4.8 ± 0.7	4.5 ± 0.6	4.7 ± 0.6	<0.001
Glucose 120 minutes (mmol/L)	6.5 ± 2.4	6.8 ± 1.3	not assessed	0.16
Fasting insulin (pmol/L)	63.7 ± 44.6	44.3 ± 20.6	59.7 ± 62.9	<0.001
HOMA-IR	2.05 ± 1.52	1.29 ± 0.68	1.77 ± 1.82	<0.001
hsCRP (µg/L)	3.1 ± 3.7	1.7 ± 1.6	2.7 ± 2.6	<0.01
HMW Adiponectin (µg/L)	4.1 ± 2.2	4.7 ± 2.7	4.9 ± 2.8	<0.01
Leptin (µg/L)	43.8 ± 25.2	20.9 ± 16.1	25.9 ± 20.3	<0.001
RANTES (µg/L)	47.6 ± 19.3	45.6 ± 22.6	39.8 ± 24.1	0.28
MCP-1 (ng/L)	82.1 ± 32.6	84.5 ± 23.4	89.1 ± 25.5	0.5
Chemerin (µg/L)	77.6 ± 25.7	64.8 ± 20.2	75.7 ± 21.1	<0.01
LBP (µg/L)	27.3 ± 3.3	25.8 ± 3.5	28.0 ± 3.5	<0.01
Calprotectin (µg/L)	424.9 ± 560.3	342.9 ± 351.6	142.9 ± 132.9	0.85

The VLCD resulted in a significant decrease in body weight (−6.9 ± 1.9 kg). This corresponded to a decrease in BMI (−2.5 ± 0.7 kg/m²), lean mass (−2.7 ± 1.6 kg), and total body fat mass (−4.2 ± 1.6 kg). In addition, waist circumference (−5.7 ± 5.5 cm) and hip circumference (−4.5 ± 3.7 cm) were significantly decreased. RMR did not change significantly during the 4 weeks of dietary intervention (Table 1).

During the two weeks following the intervention, participants regained on average 250 ± 1.5 g body weight (P = 0.47). Waist circumference (P = 0.44) and hip circumference (P = 0.64) did not change significantly during this post-study period. However, lean mass (P = 0.01) significantly increased and fat mass (P = 0.02) significantly decreased during the last two weeks.

### Caloric restriction improved metabolic and inflammatory markers transiently

Total cholesterol, high-density lipoprotein cholesterol (HDL-c), low-density lipoprotein cholesterol (LDL-c), and triglycerides decreased significantly following VLCD (Table 1). During the subsequent two weeks, these parameters increased again (P < 0.001 each). In addition, we observed a significant (P = 0.03) increase in nonesterified fatty acids (NEFA) during VLCD. During the subsequent two weeks, NEFA decreased significantly (P < 0.0001). Regarding glucose metabolism, fasting glucose (from 4.8 ± 0.7 mmol/L to 4.5 ± 0.6 mmol/L), plasma insulin (from 63.7 ± 44.6 pmol/L to 44.3 ± 20.6 pmol/L), and HOMA-IR index (from 2.05 ± 1.52 to 1.29 ± 0.68) were significantly lower (P < 0.001 each) after VLCD compared to baseline. During the last two weeks of the study (after returning to the habitual diet), blood glucose significantly increased (P = 0.04), whereas insulin levels (P = 0.11) and HOMA-IR did not change significantly (P = 0.16). Post-glucose concentrations during the glucose tolerance test did not change significantly before and after VLCD (P = 0.16, Table 1).

The hypocaloric diet induced a decrease in the inflammation marker hsCRP (Table 1). HMW adiponectin increased and leptin decreased (P < 0.001 each) after VLCD. The chemokines regulated on activation normal T cell expressed and secreted (RANTES) and monocyte chemoattractant protein-1 (MCP-1) did not change significantly during the whole study. Chemerin, considered to be a marker of inflammation and to be associated with obesity and the metabolic syndrome^23,24^, showed a significant decrease after caloric restriction (P < 0.01). LBP levels, a marker for translocation of cell-wall components from Gram-negative bacteria, significantly decreased after intervention (P < 0.01) and increased again during follow-up (P < 0.0001). Fecal calprotectin did not decrease significantly after VLCD (P = 0.79).

### Markers of paracellular gut permeability decreased during VLCD

Table 2 summarises the results obtained using different approaches for measurement of gut permeability. In brief, paracellular gut permeability markers, including lactulose, polyethylene glycol (PEG) 1500 (PEG_25_, PEG_30_, PEG_35_, and PEG_40_), and zonulin were consistently decreased after caloric restriction.

**Table 2:** Gut permeability parameters at three different time point. Data are presented as means ± standard deviation. P-value for differences between time points before and after VLCD was determined using paired Student’s test.

	n	before VLCD	after VLCD	14d after VLCD	p – value
		t1	t2	t3	t1−t2
Sucrose (urine recovery in %)	18	0.17 ± 0.12	0.10 ± 0.06	0.73 ± 0.94	0.01
Mannitol (urine recovery in %)	18	12.35 ± 5.38	9.30 ± 4.99	14.66 ± 9.16	0.17
Lactulose (urine recovery in %)	18	0.26 ± 0.18	0.15 ± 0.10	0.32 ± 0.32	0.01
Sucralose, urine recovery in %	18	1.09 ± 1.52	0.26 ± 0.48	1.30 ± 1.79	0.02
PEG_9_ (urine recovery in %)	20	16.8 ± 10.34	12.4 ± 7.29	16.6 ± 12.7	0.03
PEG_11_ (urine recovery in %)	20	16.5 ± 15.15	7.6 ± 5.57	17.3 ± 19.5	0.01
PEG_13_ (urine recovery in %)	20	4.2 ± 3.59	2.14 ± 1.25	4.2 ± 4.1	<0.01
PEG_25_ (urine recovery in %)	20	0.38 ± 0.33	0.17 ± 0.15	0.30 ± 0.28	<0.001
PEG_30_ (urine recovery in %)	20	0.22 ± 0.17	0.11 ± 0.08	0.15 ± 0.12	<0.001
PEG_35_ (urine recovery in %)	20	0.20 ± 0.18	0.08 ± 0.09	0.15 ± 0.20	<0.001
PEG_40_ (urine recovery in %)	20	0.24 ± 0.24	0.10 ± 0.11	0.16 ± 0.19	<0.01
PEG_70_ (urine recovery in %)	20	0.1 ± 0.08	0.04 ± 0.08	0.04 ± 0.06	0.02
PEG_80_ (urine recovery in %)	16	0.04 ± 0.05	0.02 ± 0.04	0.02 ± 0.04	0.09
Plasma Zonulin (µg/L)	20	58.4 ± 21.7	47.45 ± 11.88	54.34 ± 14.75	<0.01

Gut permeability was first measured using a mixture of four different sugars. Sucrose, used as a proxy for gastroduodenal permeability, significantly decreased after caloric restriction (P = 0.012). Two weeks after intervention, sucrose increased significantly again (P = 0.006). In contrast, mannitol, a marker for intestinal permeability, did not change significantly during the 28 days of caloric restriction (P = 0.162). However, after the post- intervention phase, its percental urine recovery was significantly increased (P < 0.001). Lactulose, used as a surrogate to monitor tight junction fitness significantly decreased during intervention (P = 0.01), while it relapsed afterwards (P < 0.01). Also, sucralose, used as colonic permeability marker, significantly decreased during VLCD (P = 0.018) and increased again after caloric restriction (P = 0.019).

In addition to sugar translocation, we used PEGs translocation as an additional measure of gut permeability. The mixture of the different molecular weights of PEG enabled size-dependent assessment of permeability properties of the mucosa. Translocation of PEG M_r_ 400 was significantly decreased after VLCD (Table 2). In the 2-week follow-up, these low molecular weight PEGs returned back to baseline levels. All larger homologues of different chain lengths (PEG_25_, PEG_30_, PEG_35_, and PEG_40_) significantly decreased after caloric restriction (P < 0.01 for each measurement), but remained stable during the subsequent two weeks. Similarly, PEG_70_ and PEG_80_ significantly decreased during the 28 days of caloric restriction and remained stable thereafter (Table 2).

Finally, zonulin was used as another marker of paracellular gut permeability^25^. Plasma concentration at baseline declined significantly after the 4-week hypocaloric intervention (P < 0.01). After the subsequent two weeks, zonulin levels returned to baseline values (P < 0.01) (Table 2).

### Effect of VLCD on adipokine expression and fat cell size

**Figure 1:**
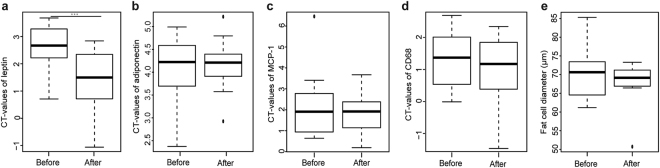
(**a**–**d**) Boxplots based on cycle threshold values (CT) of adipose tissue biopsies from 18 participants before and after the 4-week caloric restriction. (**e**) For fat cell diameters, data of 8 participants were available. ***P < 0.001.

In the whole study population, gene expression in subcutaneous adipose tissue was assessed by qPCR and showed a significant down-regulation of leptin after VLCD in comparison to baseline (P = 0.001). In contrast, the expression of adiponectin, MCP-1, and cluster of differentiation 68 (CD68) were not significantly different before and after the hypocaloric intervention (Fig. 1)

From a subsample of participants, fat cell size from para-umbilical subcutaneous adipose tissue biopsies was measured before and after VLCD (Fig. 1). Adipocyte size before VLCD was 70.5 ± 7.7 μm (n = 8) and decreased to 67.3 ± 7.1 μm (n = 8) after caloric restriction without reaching significance (P = 0.38). Mean adipocyte surface area before VLCD was 4,478 ± 997 μm^2^ and decreased to 4,020 ± 773 μm^2^ (n = 8, P = 0.31).

### Intervention triggered individual- specific changes in fecal microbiota profiles

**Figure 2:**
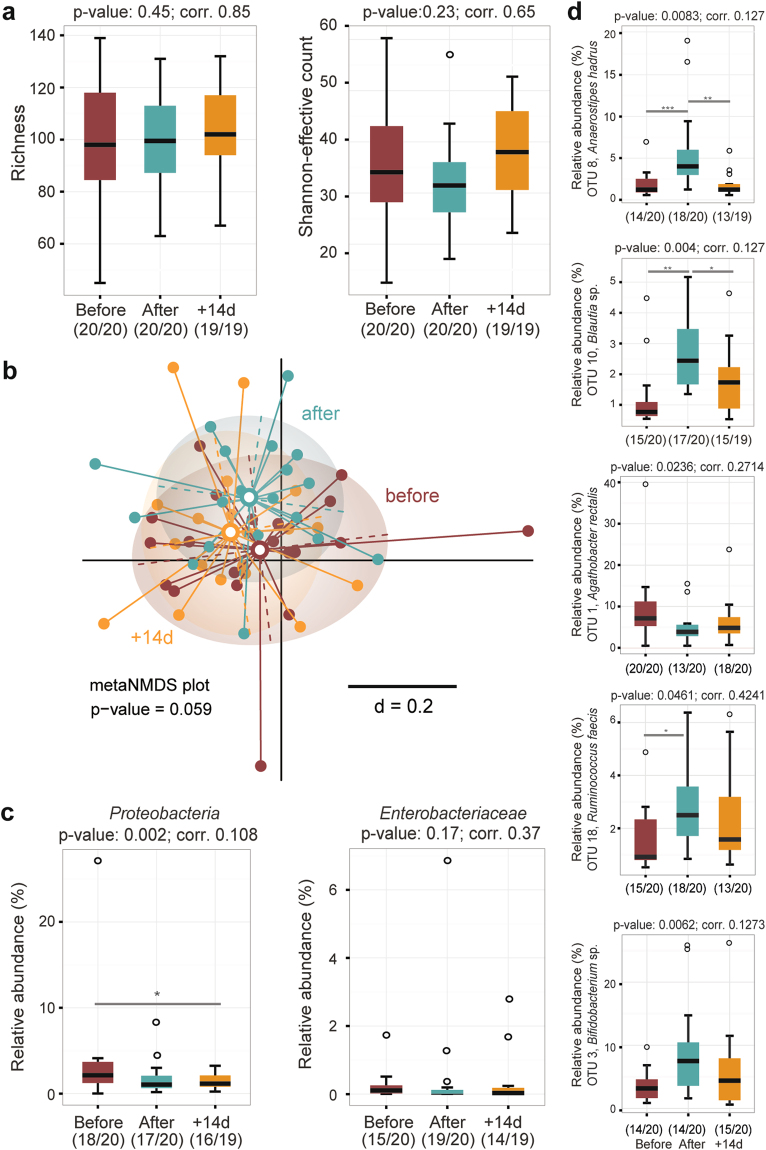
Fecal microbiota analysis by 16 S rRNA gene amplicon analysis. (**a**) Diversity within samples (*alpha*-diversity) was estimated by species richness and Shannon-effective counts. (**b**) meta nonparametric multidimensional scaling plot of phylogenetic distances based on generalized UniFrac (*beta*-diversity). (**c**) Occurrence of members of the phylum *Proteobacteria* and the family *Enterobacteriaceae*, including significance before and after Benjamini-Hochberg adjustment. (**d**) Relative abundances of five dominant OTUs showing significance overtime. ***P < 0.001; **P < 0.01; *P < 0.5.

After quality- and chimera-check, a total of 815,773 sequences clustering in 235 operational taxonomic units (OTU) were analysed. Caloric restriction did not affect *alpha*-diversity (Fig. 2a). *Beta*-diversity analysis revealed marked inter-individual differences and no significantly distinct clustering according to time points (Fig. 1b). These data suggest that caloric restriction did not trigger consistent shifts in the overall phylogenetic makeup of fecal bacterial populations; microbiota profiles remained individual-specific throughout the study (Supplementary Fig. 2). The relative abundance of members of the phylum *Proteobacteria* was significantly decreased after caloric restriction. This decrease did not hold up after correction for multiple testing. No taxonomic groups within this phylum (e.g. *Enterobacteriaceae*) showed significant differences (Fig. 2c). Of the 235 OTUs detected, five showed significant differences in their relative abundances during intervention, as per explorative analysis (Fig. 2d). Three OTUs belonged to the family *Lachnospiraceae* within the phylum *Firmicutes*. OTU 8 (*Anaerostipes hadrus*, 100% sequence identity) and OTU 10 (*Blautia sp*., several hits > 97% sequence identity) showed higher relative abundance after VLCD and returned to baseline values after two weeks. Relative abundances of OTU 1 (*Agathobacter rectalis*, 100% sequence identity) decreased after caloric restriction and throughout the end of the study. Two additional OTUs outside the *Lachnospiraceae* were characterised by intervention-related increase in their relative abundances: OTU 18 (*Ruminococcus faecis*, 100% sequence identity) within the *Ruminococcaceae* and OTU 3 (*Bifidodbacterium sp*., several hits > 97% sequence identity) within the family *Bifidobacteriaceae*.

## Discussion

The purpose of our study was to assess the effect of a standardised 4-week caloric restriction on gut permeability, inflammatory markers, and fecal microbiota in obese women. As expected, the VLCD resulted in a significant decrease in body weight and fat mass as well as improvement of fasting blood glucose, insulin, HOMA-IR and lipid parameters.

The primary objective was to study whether gut permeability, which is reported to be impaired in obesity^7^, can be positively affected by caloric restriction. Our findings based on a variety of methods indicate that a standardised caloric restriction resulting in moderate weight loss significantly reduced gut permeability, in particular paracellular translocation. These data are consistent with results of a recent Chinese study reporting decreased gut permeability after a 9-week intervention diet consisting of traditional Chinese foods and prebiotics (1, 000–1,600 kcal/day)^26^. However, our study extends this observation by providing a more comprehensive dataset using a variety of methods and a precisely defined diet. It is interesting to note that improved gut barrier function was observed along the gastrointestinal tract, as we measured clear response for sucrose reflecting gastroduodenal permeability down to sucralose, which is taken up in the colon. Likewise, decreases in the translocation of PEG particles of various sizes reflecting paracellular permeability were measured in urine samples. Zonulin, a physiological modulator of intercellular tight junctions^27^, was also found to decrease during caloric restriction, but rapidly returned to baseline levels after restoring a normal diet. In conclusion, caloric restriction induced uniform and consistent changes towards decreased gut permeability.

To be able to distinguish between caloric restriction and weight loss, we studied the participants after returning to a balanced weight-maintaining diet for two consecutive weeks. The data suggest that most changes observed under the very low calorie diet disappeared, indicating that these changes were mainly due to the acute and marked caloric restriction rather than to the moderate decrease in body weight, as participants did not gain substantial weight during the two weeks of follow-up.

As expected, the plasma concentrations of the inflammation marker hsCRP were decreased after VLCD, which is in agreement with the literature^28^. We also measured circulating LBP as a surrogate marker of translocation of cell wall components from Gram-negative bacteria, a term referred to as leaky gut-associated endotoxemia^9^. Caloric restriction induced a rather modest decrease in LPB levels^29^, which is in line with the results of the gut permeability measurements and the possibly associated decrease in inflammatory markers. However, the design of the study does not allow to draw firm conclusions on the potential causal relationship between endotoxin translocation and systemic inflammation.

Plasma leptin levels decreased markedly, while HMW adiponectin increased after intervention. In contrast, levels of MCP-1 remained unchanged. One explanation could be that a mean weight loss of 7% is too small for a larger effect on obesity-related inflammation markers^30^. In addition, the changes in gene expression for selected markers in adipose tissue – with the exception of leptin – were modest, probably due to the limited weight loss. Despite the significant weight reduction, no significant decline in fat cell size of abdominal subcutaneous adipose tissue was observed. However, there a trend towards a decrease of the mean adipocyte size. Verhoef *et al*. showed that a 10% weight loss mediated a significant decline in adipocyte size^31^.

With respect to fecal microbiota profiles, the overall bacterial phylogenetic makeup was not substantially affected by caloric restriction in a consistent manner across all individuals. Moreover, we did not observe changes in *alpha*-diversity, in contrast to changes in richness reported in the context host metabolic distrubances and nutritional intervention^32,33^. Decreased relative abundances of *Proteobacteria* were observed, in agreement with other reports on their occurrence in individuals with impaired metabolic health^34,35^. Relative abundances of the species *Anaerostipes hadrus* were increased by approximately two-fold after 28 days of caloric restriction and returned to baseline values after intervention. Although data in the present study are descriptive and exploratory, and no cause-effect relationship can be established, *A*. *hadrus* was described as a butyrate-producing bacterium^36,37^, and butyrate is usually regarded as beneficial in the context of metabolic health^38,39,32^. In contrast, median relative abundances of another butyrate-producing species, *Agathobacter rectalis*, were decreased from ca. 7 to 4% after caloric restriction. Additional studies including targeted metabolite measurements will be needed to clarify the impact of caloric restriction on butyrate production in the gut.

The intervention was also associated with an increased occurrence of one OTU in each the *Ruminococcus* and *Bifidobacterium* genus, which both include degraders of complex dietary and host-derived polysaccharides^40^. Santacruz *et al*. also showed an increase in qPCR counts of *Bifidobacterium* *spp*. after weight loss (−6,9 kg) in obese adolescents following 10 weeks of caloric restriction^41^.

The strength of our study is the strict standardisation and the extensive phenotyping of the participants. In addition, an extensive array of different methods was used to characterise the impact of VLCD on gut permeability: The metabolic status was assessed by an oGTT; The inflammation status was investigated by a variety of circulating parameters as well as gene expression in adipose tissue samples. The results were further substantiated by data related to the gut microbiota, which followed an individual pattern. Additional studies are needed to better understand these heterogenous responses.

In conclusion, our data suggest that a 4-week VLCD diet triggers beneficial effects on intestinal barrier function in obese women, which rapidly disappears after returning to a normal diet. The potential causal relationship between changes in gut permeability and the changes observed for metabolic and inflammatory biomarkers as well as specific target bacteria needs to be investigated in more detail.

### Participants and Methods

The study protocol was reviewed and approved by the ethics committee of the Faculty of Medicine of the Technical University of Munich, Germany (approval no. 5499/12). The guidelines of the International Conference on Harmonization of Good Clinical Practice and the declaration of Helsinki (in the revised version of Seoul, South Korea 2008) was basis of the study. Written informed consent was obtained from all participants before inclusion into the study. The study was registered in the German Clinical Trial Register (DRKS00006210). The date of German Clinical Trial Register registration was 11^th^ June 2014.

### Study participants

Twenty female participants with a BMI ≥ 30 kg/m² were recruited in October 2013 via advertisements in the area of Munich, Germany. The participants’ eligibility was assessed with a detailed screening questionnaire including their medical history. Exclusion criteria were: BMI < 30 kg/m², male, smoking, acute infections, severe diseases (e.g. cancer), treatment with oral anticoagulants or other antithrombotic medication, intestinal surgery in the last three months, autonomous neuropathy, or inflammatory intestinal diseases.

### Intervention

**Figure 3:**
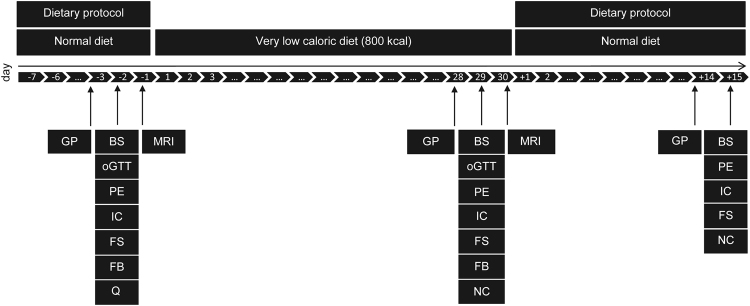
Study design. The scheme gives an overview of the timeline and different examinations performed. Abbreviations: BS, Blood sample; FB, fat biopsy; FS, fecal sample; GP, gut permeability; IC, indirect calorimetry; MRI, magnetic resonance imaging; NC, nutritional counseling; PE, physical examination; oGTT, oral glucose tolerance test; Q, questionnaire.

Figure 3 summarises the study design of this single arm intervention trial. In total, the study duration was seven weeks and was structured into three time periods. During the first period, participants were instructed to maintain their usual eating habits. Then, participants underwent a caloric restriction using a defined formula diet of 800 kcal/day for 28 days. Finally, during the two weeks following intervention, participants were instructed to follow a balanced diet of 1,800 kcal/day. Before, immediately after, and two weeks after the formula diet intervention, clinical and biochemical parameters, gut permeability, and fecal microbiota profiles were assessed. Magnetic resonance imaging (MRI) of subcutaneous and visceral fat depots and a needle aspiration of periumbilical subcutaneous adipose tissue was carried out before and after the 4 weeks of hypocaloric intervention.

### Diet protocols

The study participants were instructed to record their food consumption during the whole study period. The energy content and macronutrient composition of the diets were calculated using the OptiDiet Plus software (Version 5.1.2.046, GOE mbH, Linden, Germany).

### Anthropometric measurements

Anthropometric and clinical measurements were performed in a standardised manner between 8 and 9 am in the morning following an overnight fast. Body weight and composition were measured using the TANITA Body Composition Analyzer Type BC-418 MA III (Amsterdam, Netherlands). The resting metabolic rate (RMR) was measured using a canopy hood (COSMED Quark RMR, Fridolfing, Germany).

### Blood samples and biochemical analyses

Blood samples were collected in the fasting state. Lipid parameters (total cholesterol, LDL-c, HDL-c, triglycerides), liver enzymes (aspartate transaminase (AST), alanine transaminase (ALT), γ-glutamyltransferase (γ-GT)), creatinine, uric acid, and fasting glucose were analysed by SynLab (Munich, Germany). An additional blood sample was collected, immediately centrifuged (2, 500 *g* for 10 minutes at 20 °C) and subsequently stored at −80 °C until analysis. Leptin, chemerin, hsCRP, RANTES, MCP-1, HMW adiponectin, and LBP (all: R&D, Wiesbaden, Germany), insulin (Dako, Glostrup, Denmark), and zonulin (Immundiagnostik AG, Bensheim, Germany) were assayed in plasma using commercially available enzyme-linked immunosorbent assays (ELISA). Fecal calprotectin was measured by ELISA (CALPROLAB™ Calprotectin ELISA (HRP), FROST Diagnostika GmbH, Otterstadt, Germany). All ELISAs were performed as described by the manufacturers. NEFA were measured using a commercial test kit (Wako Chemicals GmbH, Neuss, Germany). Insulin resistance was estimated using the HOMA-IR, [HOMA-IR = insulin (μU/mL) × glucose (mmol/L)/22.5]^42^.

### Oral glucose tolerance test

Oral glucose tolerance tests (OGTT) began between 8 am and 9 am following a 12-hour overnight fast. After taking a baseline blood sample, volunteers received 75 g glucose in a volume of 300 ml (AccuCheck®-O.G.T., Roche, Mannheim, Germany). After 30, 60 and 120 minutes blood was drawn and glucose levels were determined (HemoCue Glucose 201^+^, plasma-calibrated, Ängelholm, Sweden).

### Gut permeability

Gut barrier function was assessed by means of different non-invasive tests. First, the intestinal permeability was measured via a validated sugar absorption test and a test using PEG. Both tests were performed in parallel. The principle is to measure urine excretion of orally administrated substances of different molecular masses. The tests were done directly before intervention, after intervention, and two weeks after intervention. Data are presented as percentage of ingested sugars and PEGs that were discovered in the urine, referred to as % urine recovery. Finally, the gut permeability marker zonulin was measured in blood via ELISA.

### Sugar absorption test

The sugar absorption test was performed as described by Norman *et al*.^43^. The sugars were quantified by high-performance liquid chromatography with pulsed electrochemical detection (chromatography module: 250, Dionex, Idstein, Germany)^43^.

### Polyethylene glycol absorption test

Participants received 100 ml of a PEG test solution containing 1 mg of molecular mass (M_r)_ 400 (PEG_6_-PEG_13_; mass range: 285–678 Da), 200 mg of M_r_ 1500 (PEG_20_–PEG_45_; mass range: 899–2,000 Da), 4 g of Mr 3000 (PEG_51_-PEG_90_; mass range: 2,264–3,982 Da), and 4 g of Mr 4000 (PEG_75_-PEG_115_: mass range: 3,322–5,084 Da) (Merck Darmstadt, Germany). Five hours after ingestion of the sugar test solution, the PEG test solution was drunk and urine was sampled during the following 24 hours. PEGs were analysed by liquid chromatography-mass spectrometry as described by Lichtenegger and Rychlik^44^.

### Abdominal subcutaneous adipose tissue biopsy

Abdominal subcutaneous adipose tissue specimens were obtained by needle aspiration before and after the formula diet. After washing in Krebs-Ringer-buffer, fat tissue was aliquoted into tubes containing sterilised zirkonia-glas-beads (Carl Roth, Karlsruhe, Germany), RLT-buffer (RNeasy Mini Kit, Qiagen, Hilden, Germany) and 1% (v/v) ß-mercaptoethanol (#M3148, Sigma-Aldrich, St. Louis, Missouri, USA), and then immediately frozen and stored at −80 °C. For fat histology, fat tissue was fixed in 4% buffered formalin (pH 7.4) for 24 h and finally embedded in paraffin and stored at room temperature until analysis. Determination of adipocyte size was done by using the open source cellprofiler® image analysis software (http://www.cellprofiler.org/).

### Quantitative polymerase chain reaction

The expression of specific target genes was assessed by using quantitative polymerase chain reaction (qPCR). The primer sequences used are shown in Supplemental Table 1. qPCR was performed using the Mastercycler^®^ ep realplex (Eppendorf, Hamburg, Germany). Target and housekeeping gene amplicons were detected using SYBR Green (Thermo Fisher, Scientific, Darmstadt, Germany). Importin 8 (IPO8) and peptidylprolyl isomerase A (PPIA) were used for normalization^45,46^. Each sample was run in duplicate, and negative controls without cDNA were included. The calculation of relative expression was performed by using the relative expression software tool REST© (http://rest.gene-quantification.info/)^47^.

### Fecal samples

Fecal samples were collected directly into sterile plastic containers (1,000 ml;VWR International, Munich, Germany). Participants were then asked to collect one plastic spoon at one location of the fecal material into a stool collection tube containing 8 ml DNA stabilization buffer (Stratec Molecular GmbH, Berlin, Germany). Afterwards, fecal samples were immediately frozen at −18 °C until the next visit. Participants transported the frozen fecal samples by using cooling aggregates to the lab. Finally, collection tubes were immediately stored at −80 °C.

### High-throughput 16 S ribosomal RNA gene amplicon sequencing

Samples were processed as described previously^48^. Briefly, cells were lysed by bead-beating and heat-treatment and the metagenomic DNA was purified using gDNA columns (Macherey-Nagel, Düren, Germany). Concentrations and purity were inspected using the NanoDrop^®^ system (Thermo Scientific Waltham, Massachusetts, USA). The V3/V4 region of 16 S ribosomal RNA (rRNA) genes was amplified (25 cycles) from 24 ng DNA using primers 341 F and 785 R^49^. After purification (AMPure XP system, Beckmann Coulter Biomedical GmbH) and pooling in an equimolar amount, the 16 S rRNA gene amplicons were sequenced in paired-end modus (PE275) using a MiSeq system (Illumina, Inc., San Diego, California, USA) following the manufacturer’s instructions and a final DNA concentration of 10 pM and 15% (v/v) PhiX standard library.

### Sequence analysis

Raw read files were processed based on the UPARSE approach^50^ using IMNGS^51^. Sequences were tested for the presence of chimeras using UCHIME^52^ and OTUs were clustered at a threshold of 97% sequence similarity. To avoid analysis of spurious OTUs, only those with a relative abundance > 0.5% total sequences in at least one sample were kept. SILVA (SILVA Incremental Aligner version 1.2.11)^53^ and RDP classifier (set 15; 80% confidence)^54^ were used to assign taxonomic classification to the OTUs representative sequences. Specific OTUs with differential abundances between groups were further identified using EzTaxon. Phylogenetic relationships were examined using the generalized UniFrac procedure^55^. Shannon-effective counts were determined to estimate diversity within samples (*alpha*-diversity) as described by Jost *et al*.^56^.

### Statistical analyses

Data were analysed in the R programming environment. Anthropometric and metabolic data are presented as mean ± standard deviation. *P*-values < 0.05 were regarded as statistically significant. According to data distribution, paired Student’s test was applied to assess mean differences before and after the formula diet. Rhea (v1.0.1-5) was used for analysis of fecal microbiota profiles^57^. The effect of VLCD on OTUs and taxonomic counts was tested using Friedman Rank Test for the analysis of a nonparametric randomized block design. Missing values were handled by using Skillings-Mack test. Wilcoxon Signed Rank Sum Test for matched pairs was applied for pairwise comparisons. The Benjamini-Hochberg method was used for adjustment after multiple testing. For *beta*-diversity analysis, generalized UniFrac distances were calculated using the package GUniFrac^55^.

## Electronic supplementary material

Supplementary File
