# Supplementary material for: Effect of caloric restriction on gut permeability, inflammation markers, and fecal microbiota in obese women
Source: Sci Rep. 2017 Sep 20;7:11955. doi: 10.1038/s41598-017-12109-9 (PMC5607294; doi:10.1038/s41598-017-12109-9)
Supplement: Supplementary file 1 — Supplementary File [file 41598_2017_12109_MOESM1_ESM.pdf]

Supplementary Info File - Figure 1

Title:

Effect of caloric restriction on gut permeability, inflammation markers, and fecal microbiota in obese women

Authors list: Beate Ott[1,2], Thomas Skurk[1,2], Ljiljana Hastreiter[1], Ilias Lagkouvardos[2], Sandra Fischer[2], Janine Büttner[3], Teresa Kellerer[1], Thomas Clavel[2,4], Michael Rychlik[5], Dirk Haller[2,6], Hans Hauner[*1,2,7]

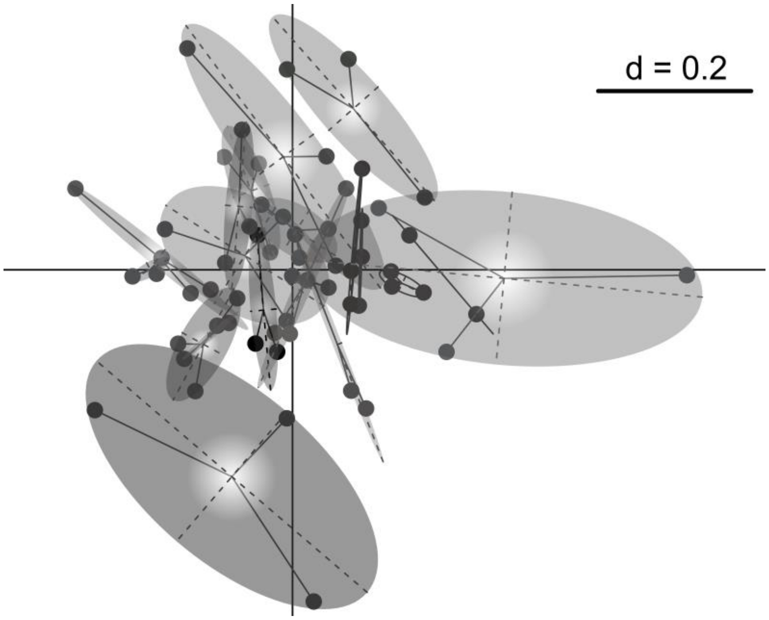

Figure 1: Individual fecal bacterial profiles before, directly after and 14d after VLCD. Ellipses show individual-specific profiles, including the three time points.

Supplemental Table 1

Title:

Effect of caloric restriction on gut permeability, inflammation markers, and fecal microbiota in obese women

Authors list: Beate Ott[1,2], Thomas Skurk[1,2], Ljiljana Hastreiter[1], Ilias Lagkouvardos[2], Sandra Fischer[2], Janine Büttner[3], Teresa Kellerer[1], Thomas Clavel[2,4], Michael Rychlik[5], Dirk Haller[2,6], Hans Hauner[*1,2,7]

Table 1: The following primer pairs were used for analysis.

Primer	Forward	Reverse
**Adiponectin**	5'-ggtgagaagggtgagaaagga-3'	3'-acactgaatgctgagcggta-5'
**Leptin**	5'-tccaagatgacaccaaaaccc-3'	3'-acgtgaagaagatcccggag-5'
**CD68**	5'-gctacatggcggtggagtacaa-3'	3'-atgatgagaggcagcaagatgg-5'
**MCP-1**	5'-gcaatcaatgccccagtc-3'	3'-ggtggtccatggaatccga-5'
**IPO8**	5'-cggattatagtctctgaccatgtg-3'	3'-tgtgtcaccatgttcttcagg-5'
**PPIA**	5'-tggttcccagtttttcatc -3'	3'-cgagttgtccacagtcagc-5'

Supplementary Info File

Title: Effect of caloric restriction on gut permeability, inflammation markers, and fecal microbiota in obese women

Authors list: Beate Ott[1,2], Thomas Skurk[1,2], Ljiljana Hastreiter[1], Ilias Lagkouvardos[2], Sandra Fischer[2], Janine Büttner[3], Teresa Kellerer[1], Thomas Clavel[2,4], Michael Rychlik[5], Dirk Haller[2,6], Hans Hauner*[1,2,7]

Separate document detailing the trial protocol

PLEASE NOTE: *This trial has been registered retrospectively.*

Trial Description

Title

Impact of caloric restriction on gut barrier integrity and metabolic diseases in human

Trial Acronym

DieGo - Dietary Intervention and Gut Barrier Function

URL of the trial

http://www.kem.wzw.tum.de/index.php?id=64

Brief Summary in Lay Language

The present study investigates the effects of a caloric restriction on gut permeability. Moreover, the project aims to examine the impact of the gut barrier function on obesity-associated metabolic diseases in human.

Brief Summary in Scientific Language

The present study investigates the effect of a caloric restriction on gut barrier integrity, leading to low-grade inflammation-induced metabolic disorders, and on obesity-associated metabolic disorders. We studied the effect of a 28-day caloric restriction (800 kcal/day) on gut permeability, gut microbiota, peripheral immune cell sub-populations and insulin resistance in 20 obese women. [Timeframe: at baseline, after a 28–day intervention period and, 14 days after intervention is terminated]

Organizational Data

- DRKS-ID: **DRKS00006210**
- Date of Registration in DRKS: **2014/06/11**
- Date of Registration in Partner Registry or other Primary Registry: **[---]***
- Investigator Sponsored/Initiated Trial (IST/IIT): **yes**
- Ethics Approval/Approval of the Ethics Committee: **Approved**
- (leading) Ethics Committee Nr.: **5499/12 , Ethik-Kommission der Fakultät für Medizin der Technischen Universität München**

Secondary IDs

Health condition or Problem studied

- Free text: **obesity-associated metabolic diseases**

Interventions/Observational Groups

- Arm 1: **20 obese women (BMI greater or equal 30 kg/m²) underwent a caloric restriction for 28 days. The dietary intervention includes a formula diet with a daily energy intake of 800kcal with additional 200g of vegetables. Before intervention, immediately after intervention and two weeks after intervention the gut permeability, changes in gut microbiota, insulin sensitivity, peripheral immune cell sub-populations, blood pressure, pulse, metabolic rate, blood parameters (fasting glucose, fasting insulin, liver function, kidney function, electrolyte, lipid profile, DNA, inflammation marker) are measured.**

Characteristics

- Study Type: **Interventional**
- Study Type Non-Interventional: **[---]***
- Allocation: **Single arm study**
- Blinding: **[---]***
- Who is blinded: **[---]***
- Control: **Uncontrolled/Single arm**
- Purpose: **Prevention**
- Assignment: **Single (group)**
- Phase: **N/A**
- Off-label use (Zulassungsüberschreitende Anwendung eines Arzneimittels): **N/A**

Primary Outcome

**gut permeability (using polyethylene glycol and sugar absorption test), changes in colon microbiota, inflammation marker in plasma as well as in peripheral immune cell sub-populations.
[Timeframe: baseline, after a 28–day intervention period, 14 days after intervention is terminated]**

Secondary Outcome

**insulin sensitivity, body composition, basal metabolic rate, blood parameters (fasting glucose, fasting insulin, liver function, kidney function, electrolytes, lipid profile, DNA, inflammation marker), blood pressure, pulse, eating behavior.
[Timeframe: baseline, after a 28–day intervention period, 14 days after intervention is terminated]**

Countries of recruitment

- DE **Germany**

Locations of Recruitment

- other **Else Kröner-Fresenius-Zentrum für Ernährungsmedizin, Freising und München**

Recruitment

- Planned/Actual: **Actual**
- (Anticipated or Actual) Date of First Enrollment: **2013/10/16**
- Target Sample Size: **20**
- Monocenter/Multicenter trial: **Multicenter trial**
- National/International: **National**

Inclusion Criteria

- Gender: **Female**
- Minimum Age: **18 Years**
- Maximum Age: **no maximum age**

Additional Inclusion Criteria

BMI >= 30kg/m²; Stable weight during the last 3 months (+/-2kg); Non-smokers; Signed informed consent

Exclusion criteria

Severe general diseases, e.g. cancer, COPD, severe heart disease (e.g. pacemaker), progressive renal failure, severe psychiatric or neurological diseases; Diabetes; Continuous treatment with oral anticoagulants or antithrombotics (except ASS 100); Malabsorption syndromes; Autonomous neuropathy; Poor compliance; lactose intolerance, sucrose intolerance, sucralose intolerance; Lack of written informed consent

Addresses

- **Primary Sponsor**

Bundesministerium für Bildung und Forschung Dienstsitz Berlin
Friedrichstraße 130 B
10117 Berlin
Germany

Telephone: **[---]***
Fax: **[---]***
E-mail: **[---]***
URL: **www.bmbf.de**

- **Contact for Scientific Queries**

Else Kröner-Fresenius-Zentrum für ErnährungsmedizinLehrstuhl für ErnährungsmedizinTechnische Universität München
Mr. Professor Hans Hauner
Gregor-Mendel-Str. 2
85350 Freising-Weihenstephan
Germany

Telephone: **08161/71-2001**
Fax: **08161/71-2097**
E-mail: **hauner at wzw.tum.de**
URL: **http://www.em-tum.de**

- **Contact for Public Queries**

Else Kröner-Fresenius-Zentrum für ErnährungsmedizinLehrstuhl für ErnährungsmedizinTechnische Universität München
Ms. Beate Ott
Gregor-Mendel-Str. 2
85350 Freising-Weihenstephan
Germany

Telephone: **08161/71-2364**
Fax: **08161/71-2097**
E-mail: **beate.ott at tum.de**
URL: **http://www.em-tum.de**

Sources of Monetary or Material Support

- **Public funding institutions financed by tax money/Government funding body (German Research Foundation (DFG), Federal Ministry of Education and Research (BMBF), etc.)**

  Bundesministerium für Bildung und Forschung Dienstsitz Berlin
  Friedrichstraße 130 B
  10117 Berlin
  Germany

  Telephone: **[---]***
  Fax: **[---]***
  E-mail: **[---]***
  URL: **www.bmbf.de**

Status

- Recruitment Status: **Recruiting complete, follow-up complete**
- Study Closing (LPLV): **2014/04/23**

Trial Publications, Results and other documents

** This entry means the parameter is not applicable or has not been set.*
